# MSC-derived exosomes attenuate cell death through suppressing AIF nucleus translocation and enhance cutaneous wound healing

**DOI:** 10.1186/s13287-020-01616-8

**Published:** 2020-05-11

**Authors:** Guifang Zhao, Feilin Liu, Zinan Liu, Kuiyang Zuo, Bo Wang, Yuying Zhang, Xing Han, Aobo Lian, Yuan Wang, Mingsheng Liu, Fei Zou, Pengdong Li, Xiaomei Liu, Minghua Jin, Jin Yu Liu

**Affiliations:** 1 Department of Toxicology, School of Public Health, Jilin University, No. 1163 Xinmin Street, Changchun, Jilin, 130021 China; 2 Department of Pathology, Jilin Medical University, Jilin, China; 3 Department of Ophthalmology, the Second Hospital of Jilin University, Changchun, China

**Keywords:** Apoptosis, Apoptosis-inducing factor, Exosomes, Mesenchymal stem cells, Poly (ADP-ribose) polymerase-1, Protease-activated receptor, Skin wound healing

## Abstract

**Background:**

Skin wounding is very common and may be slow to heal. Increasing evidence shows that exosomes derived from mesenchymal stem cells (MSCs) dramatically enhance skin wound healing in a paracrine manner. However, the mechanism underlying this phenomenon has not yet been elucidated. Thus, the objective of the present study was to identify the signaling pathways and paracrine factors by which MSC-derived exosomes promote de novo skin tissue regeneration in response to wound healing.

**Methods:**

In vitro and in vivo skin wound healing models were created by treating immortalized human keratinocytes (HaCaT) with hydrogen peroxide (H_2_O_2_) and excising full-thickness mouse skin, respectively. Exosomes were extracted from human umbilical cord Wharton’s jelly MSCs (hucMSC-Ex) by ultracentrifugation of cell culture supernatant.

**Results:**

The hucMSC-Ex treatment significantly increased HaCaT cell proliferation and migration in a time- and dose-dependent manner, suppressed HaCaT apoptosis induced with H_2_O_2_ by inhibiting nuclear translocation of apoptosis-inducing factor (AIF) and upregulating poly ADP ribose polymerase 1 (PARP-1) and poly (ADP-ribose) (PAR). The animal experiments showed that relative to hucMSCs, hucMSC-Ex attenuated full-thickness skin wounding by enhancing epidermal re-epithelialization and dermal angiogenesis.

**Conclusions:**

These findings indicated that direct administration of hucMSC-Ex may effectively treat cutaneous wounding and could be of great value in clinical settings.

## Background

The skin is the largest tissue in the body, and its main function is to protect the subcutaneous tissue. Wound healing is a complex process, and successful skin wound healing requires a series of steps, including inflammation, new tissue formation, and remodeling [1]. In addition, the migration, proliferation, differentiation, and apoptosis of skin cells play an important role [2]. The mechanism of poor wound healing in diabetes is not clear, but the causes of this terrible complication mainly include hypoxia, impaired angiogenesis, ROS injury, and neuropathy, resulting in long-term medical burden and decreased quality of life of patient role in this process [3, 4].

At present, it is believed that excessive apoptosis of cells with injury repair function, namely, dermal fibroblasts and epidermal keratinocytes, is one of the main reasons for the difficulty in healing or delayed healing of diabetic skin wounds [5, 6]. Diabetic wounds are characterized by the disorder of the wound healing process, and there is significantly prolonged [7]. The sustained inflammatory response will lead to excessive apoptosis of repair cells, such as fibroblasts, keratinocytes, or vascular endothelial cells, and then cause the wound to be difficult to heal or the healing is delayed [8, 9]. In the study of the delayed wound healing in diabetic mice, one study found that 7–14 days after the injury in the diabetic mouse wound model, there was significant healing delay compared with the control group, and the apoptosis rate of repair cells around the wound surface was significantly increased and the proliferation rate was significantly decreased [10]. Therefore, by studying the apoptotic forms of basal keratinocytes, we can deeply understand the mechanism of the difficulty in healing or delayed healing of diabetic skin wounds.

Mesenchymal stem cells (MSCs) are a rich, readily accessible source of somatic stem cells. They are easily acquired, highly expanded, and efficiently differentiated into numerous lineages of specialized cells. They are used to engineer functional organs or for in situ tissue repair and regeneration [11]. A previous study [12] showed that MSCs migrate towards tissue injury sites, transdifferentiate into specialized cells, and replace diseased, lost, aged, or apoptotic cells. Recent research has demonstrated that the rates of trans-differentiation of MSCs to specialized cells and their engraftment in injured tissues are too low to effect substantial repair and regeneration [13]. Therefore, it has become an important issue in regenerative medicine research to find new biological products or materials that can replace MSCs and clarify their regeneration and repair function and mechanism.

Exosomes are nanoscale microvesicles sprouting from various types of cells by the budding way [14]. The exosomes encapsulate nucleic acids, proteins, and lipids from the donor cells. When they are released, exosomes deliver these bioactive molecules to recipient cells such as resting stem cells in the stem cell niche or injured cells in the traumatic microenvironment [15, 16]. They then induce regeneration by activating resting stem cells or recovering the functionality of the injured cells. Exosomes have functional tissue repair and regeneration properties resembling those of the cells from which they are derived [17]. They induce none of the apparent adverse effects associated with stem cell implantation such as immunogenicity [18], malignant transformation [19, 20], or vascular obstruction [21, 22]. Thus, exosome-based therapy may be safer, easier, and more efficient for tissue repair and regeneration than stem cell-based therapy. Localized injections of exosomes originating from mesenchymal stem cells may promote skin cell proliferation and migration and angiogenesis via the wnt4 pathway or Erk1/2 signaling in animal burn or diabetic wound models [23]. Therefore, exosome-based therapy could be a promising approach to wound healing. Our group showed that direct administration of human umbilical cord mesenchymal stem cells (hucMSCs) into full-thickness skin defects of immunocompetent mice significantly enhanced cutaneous wound healing. However, transplantation of human cells into mice is xenografting, and the implanted cells were inevitably rejected by the host immune response. For this reason, hucMSCs-initiated paracrine factors play predominant roles in cutaneous wound healing. However, the mechanism by which they enhance skin wound healing remains to be elucidated [24].

To this end, we created cellular and animal models of skin defects by treating the HaCaT epidermal cell line with hydrogen peroxide (H_2_O_2_) and by excising full-thickness skin from the backs of C57 mice, respectively. We isolated exosomes from hucMSCs (hucMSCs-Ex) by ultracentrifugation and compared them with hucMSCs-conditioned medium (hucMSCs-CM), exosome-free hucMSCs-CM (hucMSCs-dp-Ex), and hucMSCs in terms of skin wound healing efficacy. Our results showed that hucMSCs-Ex significantly inhibited the nuclear translocation of apoptosis-inducing factor (AIF), the activation of poly (ADP-ribose) polymerase-1 (PARP1), and the induction of HaCaT cell apoptosis by H_2_O_2_. It also enhanced cutaneous wound healing in full-thickness skin defect animal models as did hucMSCs and hucMSCs-CM.In this study, we hypothesized that hucMSCs-Ex can promote cutaneous wound healing via the PARP-1/AIF apoptosis pathway.

## Methods

### Derivation of hucMSCs from human umbilical cord Wharton’s jelly

The present study was approved by the Ethics Committee of the School of Public Health, Jilin University, Jilin, China. The umbilical cords were obtained from healthy patients at the Affiliated Hospital of Jilin University. The donors had granted prior informed written consent. The isolation, phenotype characterization, and multipotency assay of mesenchymal stem cells (hucMSCs) from umbilical cord Wharton’s jelly were performed as previously described [24]. Briefly, hucMSCs were obtained by organ harvest and cultured in Dulbecco’s modified Eagle’s medium (DMEM, high-glucose; Invitrogen, Carlsbad, CA, USA) containing 10% (w/v) fetal bovine serum (FBS; HyClone, Melbourne, Australia), 100 U mL^−1^ penicillin/streptomycin (Sigma, Munich, Germany) and 10 ng mL^−1^ basic fibroblast growth factor (bFGF; Peprotech, London, UK) at 37 °C under a 5% CO_2_ atmosphere. Immunofluorescence staining and flow cytometry were performed on hucMSCs to evaluate the expression levels of the surface markers CD34, CD45, CD44, CD73, CD90, and CD105 (BD Biosciences, Franklin Lakes, NJ, USA). For the multipotency assay, the hucMSCs were cultured in adipogenic medium consisting of DMEM, 10% (w/v) FBS, 1 μM dexamethasone (Sigma-Aldrich Corp., St Louis, MO, USA), 0.5 mM isobuty1 methylxanthine (Sigma-Aldrich Corp., St Louis, MO, USA), 200 μM indomethacin (Sigma-Aldrich Corp., St Louis, MO, USA), and 10 μM insulin (Sigma-Aldrich Corp., St Louis, MO, USA) in osteogenic medium consisting of DMEM, 10% (w/v) FBS, 10 mM β-glycerophosphate (Alfa Aesar, Ward Hill, MA, USA), 0.1 μM dexamethasone, and 50 μM ascorbate-2-phosphate (Sigma-Aldrich Corp., St Louis, MO, USA) and chondrogenic medium consisting of DMEM, 10% (w/v) FBS, 6.25 μg mL^−1^ insulin, 10 ng mL^−1^ transforming growth factor-beta 1 (PeproTech, London, UK), and 50 nM ascorbate-2-phosphate (Sigma-Aldrich Corp., St Louis, MO, USA). Three weeks after adipogenic, osteogenic, and chondrogenic induction, intracellular lipid droplets, mineralized bone nodules, and sulfated proteoglycans were detected by Oil Red O staining, Alizarin Red-S staining, and Alcian Blue staining (Dingguo, Beijing, China) according to the manufacturers’ instructions.

### Derivation, labeling, and uptake assays of hucMSCs-Ex

Exosome isolation from MSCs was described elsewhere [25]. Briefly, hucMSCs at passage 4 were cultivated in DMEM containing 10 ng mL^−1^ bFGF and 10% (w/v) FBS deprived of exosomes by centrifugation at 120,000×*g* overnight at 4 °C. When the hucMScs reached 80% confluency, they were cultivated in DMEM containing 2% (w/v) exosome-free fetal bovine serum (FBS) for 24 h. The culture medium was then collected and centrifuged at 300×*g* at 4 °C for 10 min to pelletize the cells. The supernatant was collected, centrifuged at 16,500×*g* (Optima™ L-100XP ultracentrifuge; Beckman Coulter, Palo Alto, CA, USA) at 4 °C for 20 min then passed through a 0.22-μm filter to remove cell debris. This medium was designated conditioned medium (hucMSCs-CM). The filtrate was centrifuged at 120,000×*g* at 4 °C for 90 min. The exosomes were collected and designated hucMSCs-derived exosomes (hucMSCs-Ex). The hucMSCs-Ex were resuspended in phosphate-buffered saline (PBS) and stored at − 80 °C. The protein concentration in the hucMSCs-Ex was measured with bovine calf albumin (BCA) kit (Beyotime, Shanghai, China). The size distribution and concentration of exosomes were analyzed by nanoparticle tracking analysis using a ZetaView particle tracker from ParticleMetrix (Germany), Each NTA measurement for the different protocols for each subject was repeated in triplicate. The morphology of the hucMSCs-Ex was examined by transmission electron microscopy (TEM; FEI Tecnai 12; Philips, Amsterdam, The Netherlands). The expression levels of CD9 and CD63 (1:500, Millipore, Temecula, CA, USA) and Alix (1:1000, Abcam, USA) and TSG101 (1:500, ProteinTech, Chicago, USA) and HSP70 (1:500, SCB, USA,) in the exosomes were determined by western blot assay. The exosome-free medium was designated exosomes-deprived hucMSCs-conditioned media (hucMSCs-dp-Ex).

The hucMSCs-Ex were labeled with PKH26 (Sigma-Aldrich Corp., St. Louis, MO, USA) as previously described [26]. In brief, 2 μL PKH26 was mixed with 1 mL hucMSCs-Ex (1 μg mL^−1^) and incubated at room temperature (20–25 °C) for 25 min. Then, 1 mL of 1% (w/v) bovine serum albumin (BSA; Roche Diagnostics, Mannheim, Germany) was added to the incubation mixture to terminate labeling. PKH26-labeled hucMSCs-Ex were collected by centrifugation at 100,000×*g* at 4 °C for 2 h, washed by PBS for once, then used as a supplement in the HaCaT cell culture. The HaCaT cells were cultured with PKH26-labeled hucMSCs-Ex for 24 h, fixed with 4% (w/v) paraformaldehyde, counterstained with Hoechst 33342 (Invitrogen, Carlsbad, CA, USA), observed under a fluorescence microscope (4000B; Leica Microsystems, Wetzlar, Germany), and photographed with a microscope-mounted digital camera (DFC500; Leica Microsystems, Wetzlar, Germany).

### Cell viability, apoptosis assays, and ROS detection

Immortalized epidermal HaCaT cells were purchased from Peking Union Medical College Hospital, Beijing, China, and cultured in DMEM supplemented with 10% (w/v) FBS (HyClone, Thermo Fisher Scientific, Melbourne, Australia) and 100 U mL^−1^ penicillin/streptomycin (Sigma-Aldrich Corp., St. Louis, MO, USA) at 37 °C under a 5% CO_2_ atmosphere. For the cell viability, ROS generation, and apoptosis assays, the HaCaT cells were seeded into 96- or 6-well tissue culture plates in triplicate at a density of 5 × 10^4^ cm^−2^ and cultured for 24 h in DMEM supplemented with 10% (w/v) FBS. The culture medium was then aspirated and the cells were washed with PBS and cultured in DMEM containing 2% (w/v) FBS with or without H_2_O_2_ at a final concentration of 1 mM for another 0.5 h, 1 h, 2 h, 4 h, and 6 h. Then CCK8 (Dojindo Molecular Technologies, Tokyo, Japan), reactive oxygen species (ROS) (Millipore EMD, Billerica, MA, USA), propidium iodine-Annexin V (San Jian, Tianjin, China), and TUNEL (Promega, Madison, WI, USA) staining were performed to measure cell viability, ROS generation, and apoptosis at the indicated time points according to the manufacturers’ instructions.

HaCaT cells were seeded in a 24-well plate at a density of 5 × 10^4^ cm^−2^ and cultured overnight in DMEM containing 10% (w/v) FBS. Next day, the culture medium was aspirated and the cells were washed thrice in PBS and cultured in DMEM containing 2% (w/v) exosome-deprived FBS and 1 mM H_2_O_2_ with the PARP-1 inhibitor 3,4-dihydro-5-[4-(1-piperidinyl)butoxy]-1(2H)-isoquinolinone (DPQ) (MedChemExpress, Monmouth Junction, NJ, USA) or the broad-spectrum caspase inhibitor Z-VAD.fmk (Santa Cruz Biotechnology, Dallas, TX, USA) at the indicated dosages or at 1000 ng mL^−l^ hucMSCs-Ex for 4 h. Then propidium iodine-Annexin V staining and flow cytometry were performed to detect HaCaT cell apoptosis.

### Cell proliferation and migration assay

HaCaT cells were seeded in a 96-well plate at a density of 5 × 10^4^ cm^−2^ and cultured overnight in DMEM containing 10% (w/v) FBS. The next day, the culture medium was aspirated and the cells were washed thrice in PBS and cultured for 4 h in DMEM containing 2% (w/v) exosome-free FBS with or without 1 mM H_2_O_2_. The cells were then washed thrice in PBS and cultured for another 24 h in DMEM containing 2% (w/v) exosome-free FBS (control), hucMSCs-CM, hucMSCs-dp-Ex, or hucMSCs-Ex at the indicated concentrations or time points. Then, a CCK8 assay was run to detect HaCaT proliferation under the aforementioned culture conditions.

Transwell and cell scratching assays were implemented to determine cell migration. Briefly, HaCaT cells were seeded in the upper chamber of a Transwell (8-μm pore filters; Corning, Corning, NY, USA) according to the manufacturer’s instructions. The HaCaT cells were also seeded in 6-well plates at a density of 5 × 10^4^ cm^−2^ and cultured overnight in DMEM containing 10% (w/v) FBS. The next day, the cells were washed thrice in PBS and cultured for 4 h in DMEM containing 2% (w/v) exosome-free FBS with or without 1 mM H_2_O_2_. Then, the HaCaT cells cultured in the 6-well plates were scratched with a 200-μL micropipette tip. The HaCaT cells cultured in the Transwell and 6-well plates were washed thrice in PBS and cultured for 24 h in DMEM containing 2% (w/v) exosome-deprived FBS, hucMSCs-CM, hucMSCs-dp-Ex, or hucMSCs-Ex in 6-well plates or in 100 μL DMEM containing 2% (w/v) exosome-deprived FBS in the upper Transwell chamber. The lower Transwell chamber was filled with 600 μL DMEM containing 2% exosome-free FBS, hucMSCs-CM, hucMSC-dp-Ex, and 1000 μg hucMSCs-Ex for 24 h. Then, the Transwells were removed from the tissue culture plate, the culture medium in the upper Transwell chamber was aspirated, and the HaCaT cells in the Transwells were fixed with methanol, dried under a laminar flow hood, and stained with Hoechst 33342 (1:10,000, RT, 2 min; Life Technologies, Carlsbad, CA, USA). A cotton tab was used to remove the HaCaT cells retained in the upper Transwell chamber and the cells were washed thrice in PBS. The HaCaT cells retained in the lower Transwell chamber were photographed under a fluorescent microscope (Olympus, Tokyo, Japan). The HaCaT cells in five randomly selected view fields were analyzed by ImageJ software (NIH, Bethesda, MD, USA). The HaCaT cells cultured in 6-well plates were photographed. ImageJ was used to measure their migration under the aforementioned culture conditions. Migration rates were calculated as follows: (distance of cells at scratching time point−distance of cells 24 h post-scratching)/(distance of cells at scratching time point).

### Protein extraction and western blot assay

HaCaT cells were seeded in tissue culture plates at a density of 5 × 10^4^ cm^−2^ and cultured overnight in DMEM containing 10% (w/v) FBS. Next day, the cells were washed thrice in PBS and cultured in DMEM containing 2% (w/v) exosome-free FBS with or without 1 mM H_2_O_2_ for the indicated time intervals. Then the HaCaT cells were washed thrice in PBS and either harvested by trypsin digestion and centrifugation or left for another 24 h in DMEM containing 2% (w/v) exosome-free FBS, hucMSCs-CM, hucMSCs-dp-Ex, or hucMSCs-Ex at the indicated concentrations. Then, the HaCaT cells were washed twice in cold PBS and harvested by trypsin digestion as described above. Total, cytoplasmic, nuclear, and mitochondrial proteins from the HaCaT cells were extracted using kits (Keygentec, Nanjing, China) according to the manufacturer’s instructions. Briefly, the HaCaT cells were lysed by ultrasound in ice-cold radioimmunoprecipitation assay (RIPA) buffer containing 1 mM phenylmethylsulfonyl fluoride (PMSF) (Beyotime Biotechnology, Jiangsu, China) on ice. The lysates were centrifuged at 12,000×*g* for 20 min at 4 °C. The supernatants contained the total protein. For cytoplasmic and nuclear protein extraction, the HaCaT cells were resuspended in protein extraction buffer A [10 mM HEPES (pH 7.4), 1.5 mM MgCl_2_, 10 mM KCl, 0.5 mM DTT, and 0.1% (w/v) NP-40] then buffer B was added and cell lysates were centrifuged at 16,000×*g* and 4 °C for 5 min. The supernatant was collected, an equal volume of buffer C was added to it, and the mixture was centrifuged at 15,000×*g* for 10 min at 4 °C. The supernatant was collected and designated the cytoplasmic protein. The nuclear pellet was washed with buffer A, resuspended in buffer C [20 mM HEPES (pH 7.9), 1.5 mM MgCl_2_, 0.5 mM dithiothreitol (DTT), 25% (w/v) glycerol, 0.5 mM PMSF, 0.2 mM EDTA, and 420 mM NaCl], and centrifuged at 16,000×*g* for 10 min at 4 °C. The supernatant was then isolated. For mitochondrial protein separation, the post-centrifugation cytoplasm fraction was transferred to a new tube and the mitochondrion pellet was resuspended in buffer D (20% (w/v) glycerol, 20 mM HEPES, 10 mM KCl, 1.5 mM MgCl_2_, 1 mM EDTA, 1 mM EGTA, 1 mM DTT, 0.5 mM PMSF, 8 μg/mL aprotinin, 2 μg/mL leupeptin) and centrifuged at 15,000×*g* for 10 min at 4 °C. The pellet contained the mitochondrial protein.

### Cell viability, apoptosis assays, and ROS detection

Equal amounts of protein were separated by sodium dodecyl sulfate-polyacrylamide gel electrophoresis (SDS-PAGE) and electronically transferred to a polyvinylidene fluoride (PVDF) membrane (Millipore EMD, Billerica, MA, USA). The membranes were blocked in 5% (w/v) non-fat milk at room temperature for 1 h and incubated overnight at 4 °C with the antibodies listed in Table 1. After being washed three times in TBST (Tris-buffered saline with Tween-20), the membranes were incubated with the appropriate horseradish peroxidase-conjugated secondary antibodies for 1 h at room temperature then with ECL (enhanced chemiluminescence) reagent (Beyotime Biotechnology, Jiangsu, China). Proteins were detected with a GENE GNOME (Gene Company Ltd., Hong Kong, China). Gray intensities were measured with GeneTools software (Syngene, Frederick, MD, USA).

**Table 1:** List of antibodies used for Western blott assay

Antibody	Antibody dilution	Supplier	Catalog number
AIF	1:1000	CST*	4542s
PARP-1	1:1000	CST	9532s
PAR	2.5 μg mL^−1^	IBL**	1C-205
Calpain I	1:1000	CST	2556s
Cytochrome C	1:1000	CST	4280s
CypA	1:200	SCB***	SC-133494
Hsp70	1:500	SCB	SC-1060
Caspase-3	1:1000	CST	9664s
β-actin	1:3000	SunGene****	Km-9001
Histon3.1	1:3000	SunGene	Km-9005
COX IV	1:3000	CST	4844s

*Cell Signaling Technology, Danvers, MA, USA

**Immuno-Biological Laboratories, Fujioka, Japan

***Santa Cruz Biotechnology, Dallas, TX, USA

****SunGene GmbH, Gatersleben, Germany

### Immunofluorescence staining

Immunofluorescence staining was performed on HaCaT cells as previously described [24]. Briefly, HaCaT cells were cultured in DMEM containing 10% (w/v) FBS in 24-well tissue culture plates. When the cells reached 70% confluence, they were incubated for 4 h in DMEM containing 2% (w/v) exosome-free FBS with or without 1 mM H_2_O_2_. Alternatively, the HaCaT cells were pretreated for 1 h with DPQ (30 μM mL^−1^) and Z-VAD.fmk (100 μM mL^−1^) or hucMSCs-CM, hucMSCs-dp-Ex, and hucMSCs-Ex then cultured with 1 mM H_2_O_2_ for another 4 h. The cells were then washed twice in PBS, fixed in 4% (w/v) PFA/PBS, and permeabilized in 1% (w/v) Triton X-100 followed by incubation with anti-PAR, anti-AIF, and anti-cyt C antibodies (Table 1) overnight at 4 °C. The cells were then washed and incubated with Alexa Fluor 546-conjugated donkey anti-goat IgG (1:800 dilution; Invitrogen, Carlsbad, CA, USA) or Alexa Fluor 488-conjugated anti-mouse IgG (1:400 dilution; Cell Signaling Technology, Danvers, MA, USA) at room temperature for 1 h. The cells were then extensively washed in PBS, counterstained with Hoechst 33342 (1:10,000, RT, 2 min; Life Technologies, Carlsbad, CA, USA) and observed under a fluorescence microscope (DMI4000B; Leica Microsystems, Wetzlar, Germany) and photographed with a microscope-mounted digital camera (DFC500; Leica Microsystems, Wetzlar, Germany). The percentage of positive cells in five random view fields was evaluated with ImageJ software (NIH, Bethesda, MD, USA).

### Full-thickness skin defects and mouse cutaneous wound healing

All animal experiments were conducted in compliance with the guidelines approved by the China Association of Laboratory Animal Care and the Institutional Animal Care Committee. Balb/C mice (male; 20–25 g) were purchased from Fukang Animal Breeding Center, Beijing, China, and maintained at the Institutional Animal Center of Jilin University, Jilin, China. The mice were acclimated to their environment for 1 week. After acclimation, a full-thickness excisional wound (0.8 cm × 0.8 cm) was made with surgical scissors on the dorsal skin of each mouse. The mice were randomly divided into six groups, each of which comprised 10 mice. The corners of each wound margin were subcutaneously injected with 10^6^ hucMSCs 100 μL^−1^ PBS (hucMSCs group), 100 μg hucMSCs-Ex 100 μL^−1^ PBS (hucMSCs-Ex group), 100 μL PBS (PBS group); 100 μL hucMSCs-CM (hucMSCs-CM group), 100 μL hucMSC-dp-Ex (hucMSCs-dp-Ex group), or nothing (sham group). The wounds were wrapped with a single layer of oil gauze covered by three layers of cotton gauze as previously described.

### Gross inspection and H&E and immunofluorescence staining

At days 7 and 14 after wounding, photographs of the wound sites were taken for gross inspection of wound closure. The outline along the wound margin was depicted with transparent film and the wound closure rate was calculated as follows:

((original wound area−new wound area)/original wound area) × 100.

The mice were sacrificed by anesthetic overdose and skin specimens including wounds and neighboring tissues were collected, fixed with 10% (w/v) buffered formaldehyde/PBS, embedded in paraffin, sectioned at a 5-μm thickness in the center of the wound, and stained with hematoxylin and eosin (H&E). The tissue sections were observed and photographed under a microscope. Epidermal tissue subtended by hair follicle-free dermis was designated newly formed epidermis. The histological wound healing rate was calculated as follows:

(length of newly formed epidermis)/(length of newly formed epidermis + non-healed epidermis) 

Immunofluorescence staining was performed to detect epidermal formation, dermal angiogenesis, and scar tissue in the wounded skin. Briefly, skin section slides were deparaffinized, rehydrated, and blocked in 1% (w/v) BSA/PBS at room temperature for 30 min. The slides were incubated with rat anti-mouse primary antibody against CD31 (1:100; Cell Signaling Technology, Danvers, MA, USA), cytokeratin 10 (1:100; Abcam, Cambridge, MA, USA), or α-SMA (1:200; Cell Signaling Technology, Danvers, MA, USA) at 4 °C overnight then washed thrice in PBS. The slides were incubated with Alexa Fluor-488/555-conjugated anti-mouse secondary antibody at room temperature for 30 min and counterstained with Hoechst 33342 (Invitrogen, Carlsbad, CA, USA) to track the nuclei. The slides were observed and photographed under a fluorescence microscope fitted with a digital camera (Leica, DFC500, Wetzlar, Germany). Five randomly selected fields per tissue section were observed at × 400. Three sections were chosen from three mice per group. Image-Pro Plus (Media Cybernetics, Rockville, MD, USA) was used to determine the average optical densities for CD31 and α-SMA expression. Five randomly selected fields were examined per group at each time point and used to calculate the average optical density per unit area.

### Statistical analysis

Statistical analysis was performed using SPSS v. 17.0 (IBM Corp., Armonk, NY, USA). Data are means ± standard deviation (SD) for ≥ 3 independent experiments. Multiple group comparisons were made by one-way ANOVA. Paired group comparisons were made with Student’s *t* test. *P* < 0.05 was considered statistically significant.

## Results

### Derivation of exosomes from hucMSCs

**Fig. 1:**
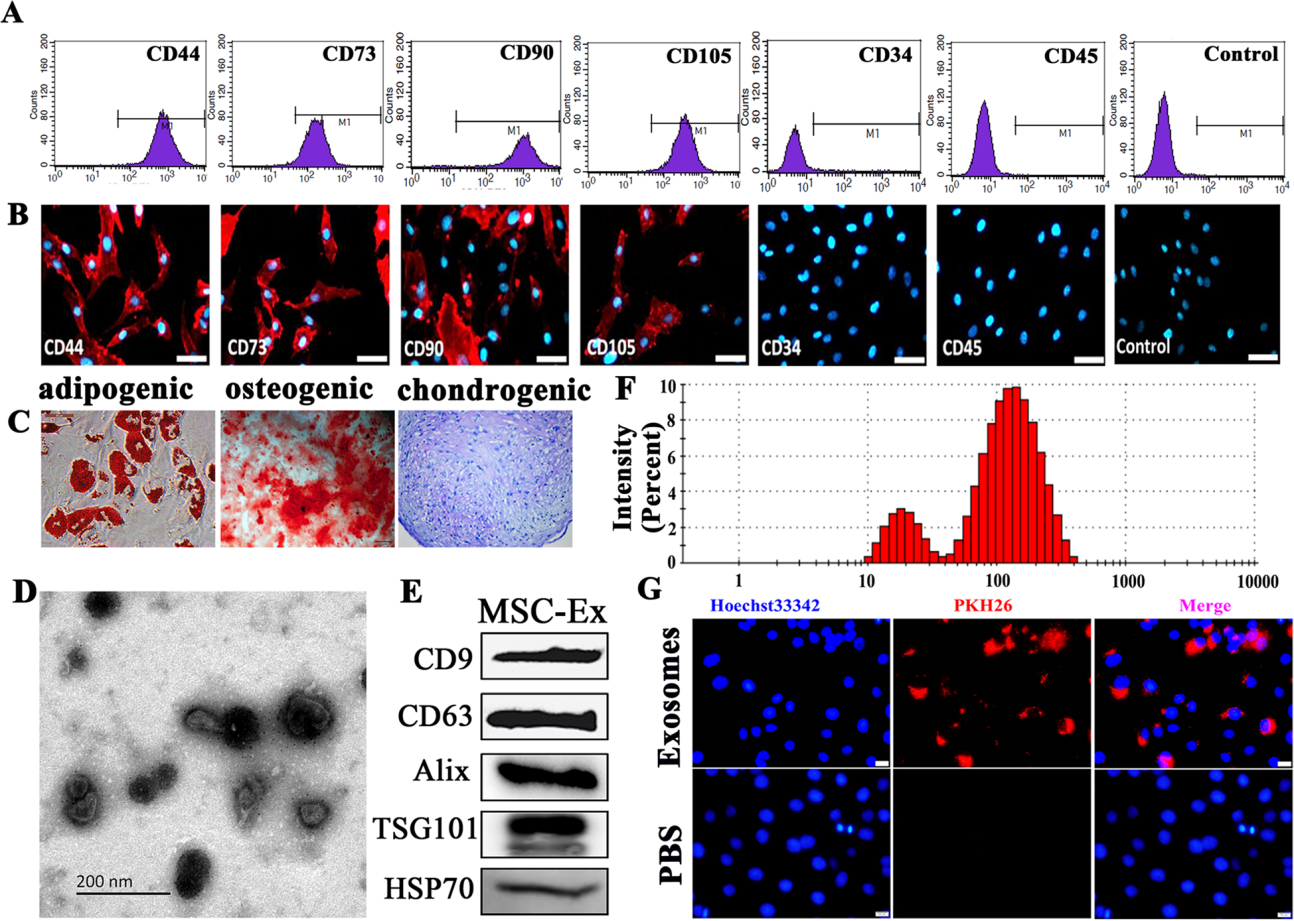
Characterization of human umbilical cord mesenchymal stem cells (hucMSC) and hucMSC-derived exosomes (hucMSC-Ex). **a**, **b** Low cytometry and immunofluorescence of phenotypic markers of MSCs. The hucMSC were positive for CD44, CD73, CD90, and CD105 and negative for CD34 and CD45. Scale bar = 200 μm. **c** Cell lineage-induced differentiation. Adipogenic, osteogenic, and chondrogenic differentiations were analyzed by Oil-Red-O, Alizarin Red, and Toluidine Blue staining, respectively. Scale bar = 100 μm. **d** Representative TEM micrographs of purified spheroid hucMSC-Ex. Scale bars = 200 nm. **e** Western blot indicated positive CD9, CD81, Alix, TSG101, and HSP70 protein expression in hucMSC-Ex. **f** Size distribution of hucMSC-Ex. **g** The PKH26-labeled exosomes (red) can go into HaCaT cell (blue)

HucMSCs were obtained from the culture of human Wharton’s jelly tissues. They were spindle-shaped and highly expressed the MSC surface markers CD44, CD73, CD90, and CD105 (Fig. 1a, b). Under the appropriate culture conditions, they differentiated into adipocytes, osteoblasts, and chondrocytes as indicated by intracellular lipid droplet formation (Oil Red O staining), mineralized bone nodules (Alizarin Red-S staining), and sulfated proteoglycans (Alcian Blue staining), respectively (Fig. 1c). The exosomes were derived from hucMSC culture containing 2% (w/v) exosome-deprived FBS by ultracentrifugation. TEM revealed that the vesicles from hucMSCs with characteristic cup-shaped morphology were observed (Fig. 1d). The presence of exosomal marker proteins CD9, CD63, Alix, TSG101, and HSP70 was detected by Western blot (Fig. 1e). The particle size distribution of vesicles displayed about 85% range from 20 to 200 nm by nanoparticle tracking analysis (Fig. 1f).

To detect hucMSC-Ex internalization by HaCaT, hucMSC-Ex was labeled with the fluorescent dye PHK26. The PKH26-labeled exosomes were incubated with HaCaT cells. Within 24 h, the fluorescent label was detected under the fluorescence microscope in the cytoplasms and nuclei of the HaCaT cells. Thus, the hucMSc-Ex was absorbed into the HaCaT cytoplasms and nuclei (Fig. 1g).

### Establishment of HaCaT apoptosis model induced by H_2_O_2_

**Fig. 2:**
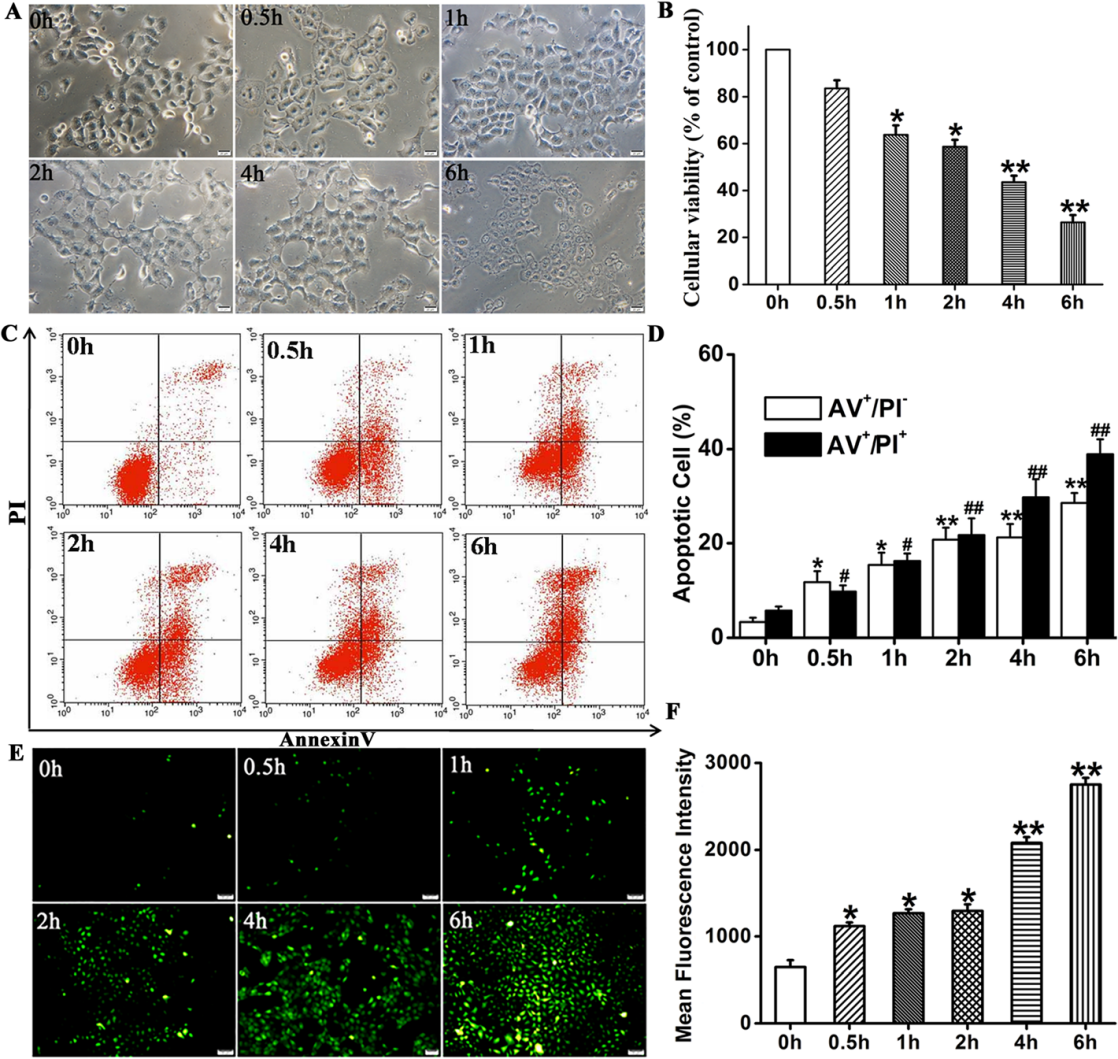
Effects of H_2_O_2_ on HaCaT according to CCK-8 assay and Annexin V-PI and ROS staining. **a** Morphological changes in HaCaT cells treated with 1 mM H_2_O_2_ at various times. **b** HaCaT cells treated with 1 mM H_2_O_2_ at different times followed by viability assay with CCK-8. Histogram shows H_2_O_2_ decreased HaCaT cell viability in a time-dependent manner. **c** Annexin V-PI staining combined with flow cytometry assay to detect apoptosis of HaCaT cells treated with 1 mM H_2_O_2_ at various times. **d** Histogram shows that apoptosis increased with induction time. **e, f** Analysis of intracellular reactive oxygen species (ROS) levels by DCF-DA assay. Histogram show that fluorescence intensity increased with induction time. Compared to 0h: ^*^P < 0.05; ^#^*P* < 0.05. Data are representative of three independent experiments (means ± SD)

To generate a skin cell damage model, HaCaT cells were treated with 1 mM H_2_O_2_ for ≤ 6.0 h. Phase-contrast microscopy showed that over time the HaCaT cell morphology changed from cobblestone to polygonal and the cells detached from the tissue culture plates (Fig. 2a). The CCK8 assay showed that HaCaT cell viability markedly decreased in a time-dependent manner from 100% to 43.56 ± 2.73% after 4 h (*P* < 0.01) and 26.7 ± 3.08% after 6 h (*P* < 0.01) following treatment with 1 mM H_2_O_2_ (Fig. 2b).

Consistent with the CCK8 assay, Annexin V-PI staining combined with flow cytometry assay showed that the proportions of cells increased with time from 5.68 ± 0.87% at time 0 to 29.75 ± 2.8% at 4 h (AV^+^ and PI^+^; *P* < 0.01) and from 3.32 ± 0.86% at time 0 to 21.23 ± 2.84% at 4 h (AV^+^/PI^-^; *P* < 0.01) (Fig. 2c, d). These values were significantly higher than those for the control group.

Flow cytometry disclosed that the ROS fluorescence intensity increased with time from 674.39 ± 78.3 at time 0 to 2750.62 ± 77.6 at 4 h (*P* < 0.01) (Fig. 2e, f).

### H_2_O_2_-induced HaCaT cell death involved spatiotemporally dependent, and caspase-dependent- and caspase-independent cell death

**Fig. 3:**
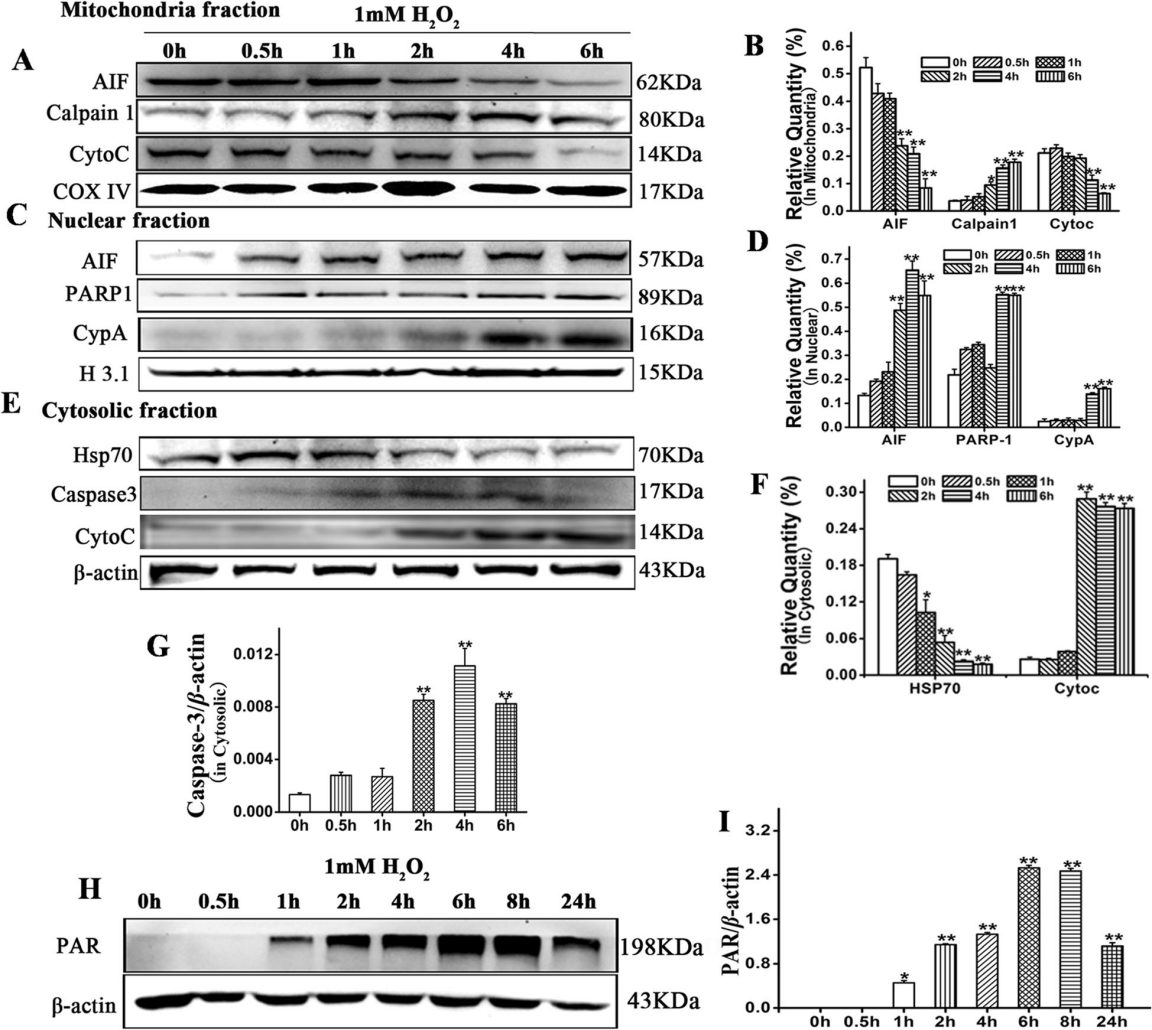
Changes of apoptotic proteins in HaCaT after H_2_O_2_ treatment. **a** Protein changes in mitochondria. **b** Compared to 0h, AIF and cyt C were downregulated and calpain 1 was upregulated following H_2_O_2_induction. **c** Protein changes in the nuclei. **d** Compared to 0 h, AIF, PARP-1, and CypA were upregulated following H_2_O_2_induction. **e** Protein changes in the cytosol. **f, g** Compared to 0 h, Hsp70 was downregulated while caspase-3 and cyt C were upregulated. **h** PAR changes in the cytosol. **i** Compared to 0 h, PAR was upregulated, reaching plateau at 6-8 hour after H_2_O_2_ treatment. Relative to 0 h: ^*^P < 0.05 and ^**^P < 0.01. Data are representative of three independent experiments (means ± SD)

H_2_O_2_-induced cell death is mitochondrial-mediated apoptosis. Caspase-dependent- and caspase-independent cell death participate in mitochondrial cell death. To determine which type of cell death affects H_2_O_2_-treated HaCaT cells, we measured the expression levels of the proteins associated with mitochondrial-mediated caspase-dependent- and caspase-independent cell death. After H_2_O_2_ treatment, expression of apoptosis-inducing factor (AIF) and cytochrome C significantly decreased in HaCaT cell mitochondria over time (*P* < 0.01) (Fig. 3a, b). AIF, cleaved PARP-1, and CypA were significantly upregulated in the HaCaT cell nuclei over time (Fig. 3c, d). Cytochrome C and cleaved caspase-3 increased in the cytosolic fraction whereas HSP70 dramatically decreased there over time (Fig. 3e–g). A whole-cell lysate assay showed that PAR accumulated in H_2_O_2_-treated HaCaT cells over time (Fig. 3h, i). The aforementioned data suggest that H_2_O_2_-induced HaCaT cell death is mitochondrially mediated and participates in caspase-dependent- and caspase-independent apoptosis. Caspase-independent cell death occurred earlier than caspase-dependent cell death.

### AIF nuclear translocation and PARP-1 activation are the predominant causes of H_2_O_2_-induced HaCaT cell death

**Fig. 4:**
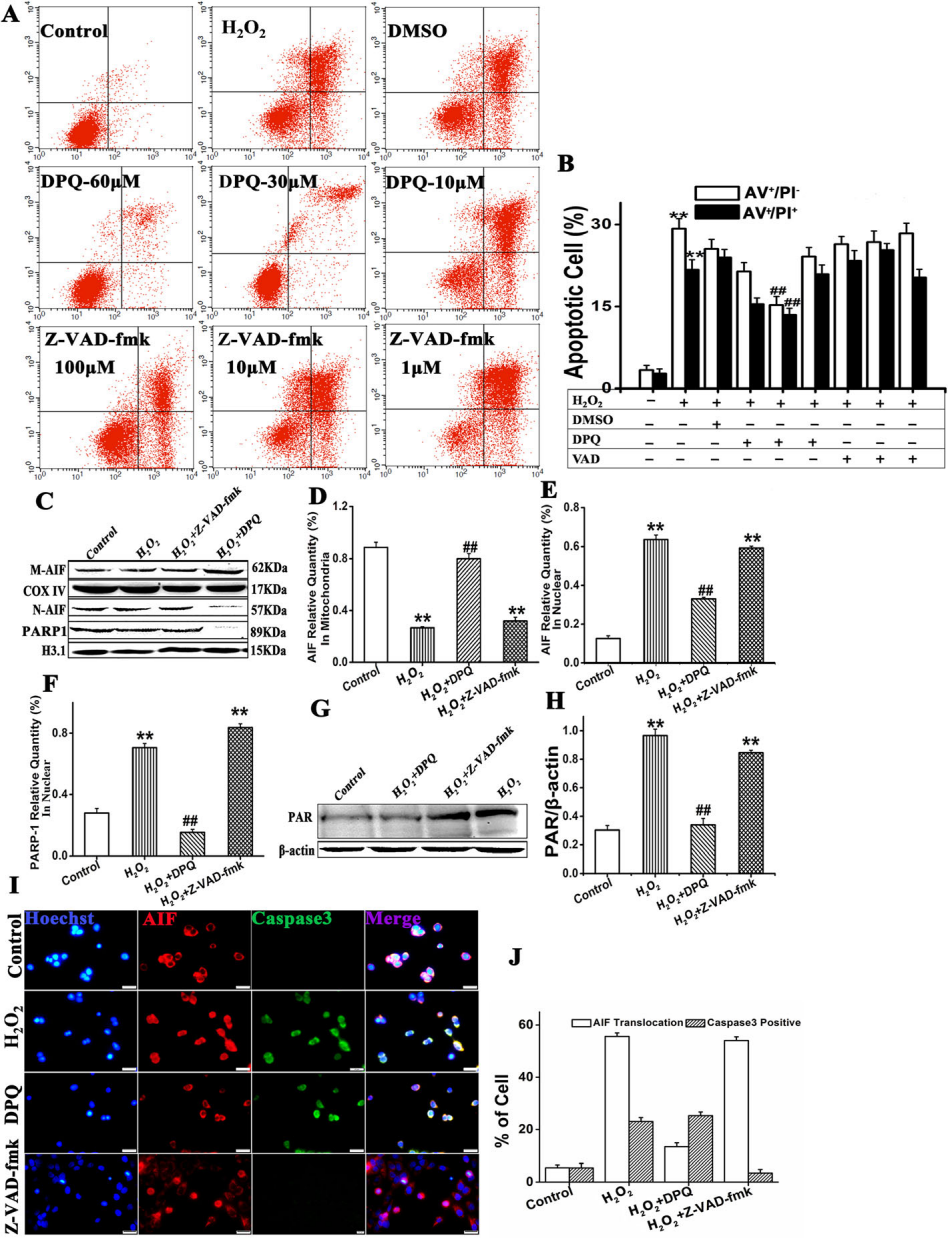
Caspase inhibitors failed to block AIF translocation in HaCaT cells treated with H_2_O_2_. **a** Annexin V-PI combined with flow cytometry was used to detect apoptosis in HaCaT cells treated with DPQ and Z-VAD-fmk. **b** PI- and AnnexinV-positive cells had lower expression levels than the H_2_O_2_ group(^#^*P* < 0.01) but not the Z-VAD-fmk treatment. **c–f** Western blot of AIF translocation by nuclear fractionation in HaCaT cells 4 h after H_2_O_2_ treatment with 30 μM DPQ or 100 μM Z-VAD-fmk. Mitochondrial AIF was upregulated in the DPQ group but downregulated in the Z-VAD-fmk group. Nuclear AIF and PARP-1 were downregulated in the DPQ group. **g**, **h** PAR in the whole protein was downregulated for the DPQ group but upregulated for the Z-VAD-fmk group. Relative to the control: ^**^P < 0.01; compared to H_2_O_2_: ^##^*P* < 0.01. Data are representative of three independent experiments (means ± SD). **i**, **j** Fluorescence microscopy of AIF and cleavedcaspase-3 for the various treatments. Scale bar = 200 μm

To determine which type of apoptosis plays a predominant role in H_2_O_2_-induced HaCaT cell death, we pretreated HaCaT cells with the broad-spectrum caspase inhibitor Z-VAD.fmk and the PARP-specific inhibitor DPQ. Annexin V/PI staining combined with flow cytometry disclosed that 30 mM DPQ significantly reduced H_2_O_2_-induced HaCaT cell apoptosis. The reductions were 15.23 ± 2.84% vs. 29.32 ± 0.86% (AV^+^ and PI^+^; *P* < 0.05). And 13.45 ± 2.85% vs. 23.68 ± 0.8% (AV^+^/PI^-^; *P* < 0.05). In contrast, 1–100 μM Z-VAD-fmk had no significant protective effects against H_2_O_2_-induced HaCaT cell apoptosis (Fig. 4a, b).

The western blot assay showed that DPQ pretreatment significantly reduced nuclear AIF and PARP/1 expression in H_2_O_2_-treated HaCaT cells. However, no significant reduction in mitochondrial AIF expression was observed. In contrast, Z-VAD-fmk pretreatment significantly downregulated mitochondrial AIF but upregulate nuclear AIF and PARP/1 in H_2_O_2_-treated HaCaT cells. DPQ pretreatment significantly reduced cytosolic PAR accumulation whereas Z-VAD-fmk pretreatment had no significant influence on cytosolic PAR expression in H_2_O_2_-treated HaCaT cells (Fig. 4c–h).

Fluorescence microscopy revealed that AIF was translocated into the nuclei of H_2_O_2_- and H_2_O_2_ + Z-VAD-fmk HaCaT cells but not into those of the H_2_O_2_ + DPQ or control HaCaT cells. PARP/1 inhibition by DPQ in H_2_O_2_-induced HaCaT cells did not downregulate cleaved caspase-3. (Fig. 4i, j).

### HucMSC-Ex markedly attenuated H_2_O_2_-induced HaCaT cell death in a dose- and time-dependent manner

**Fig. 5:**
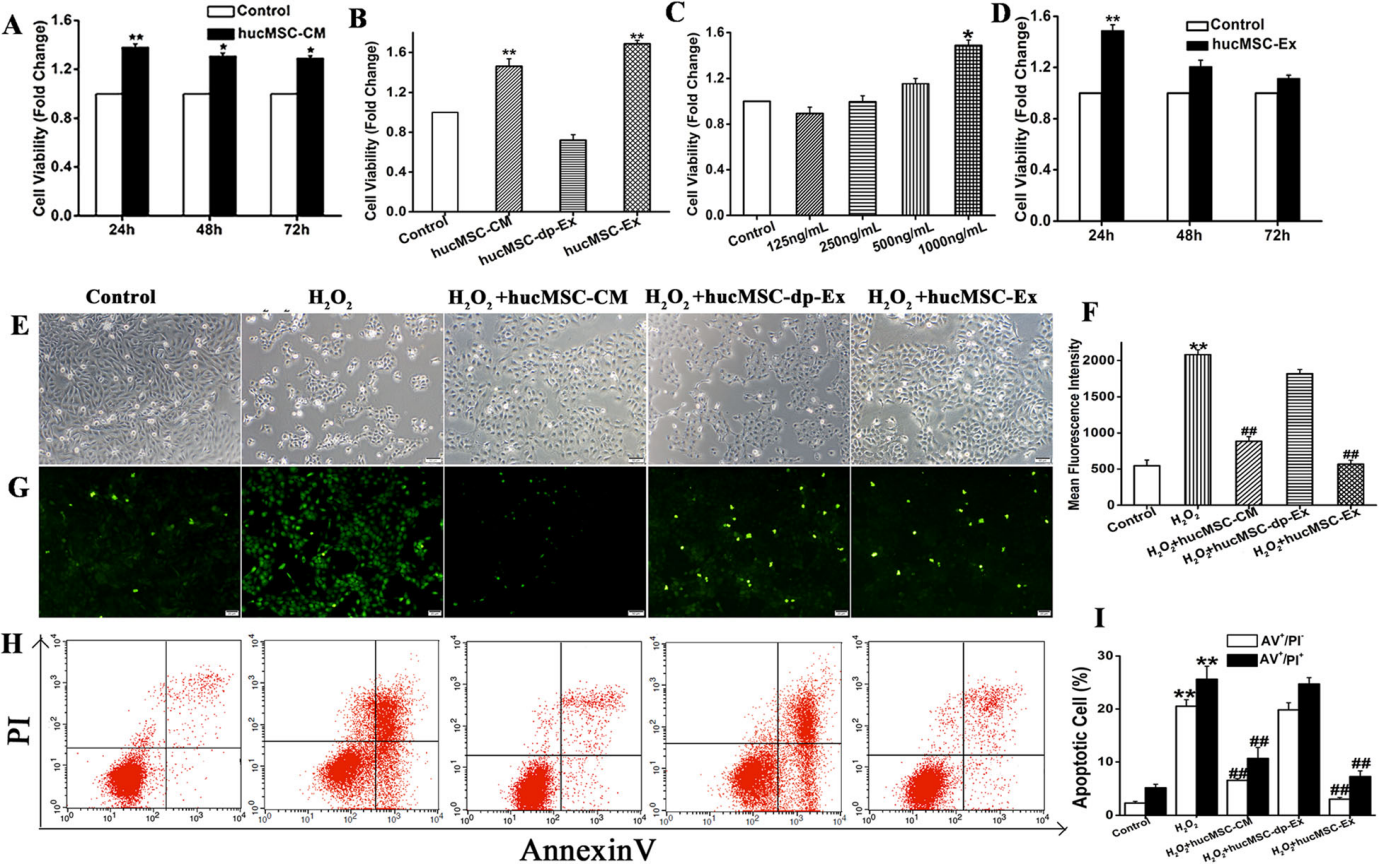
HucMSC-Ex inhibited H_2_O_2_-induced apoptosis and promoted proliferation of HaCaT cells. **a** The CCK-8 assay showed that the viability of the cells in the hucMSC-CM group was higher than that for the cells in the PBS group. **b** HaCaT cells were treated with H_2_O_2_ then cultured with hucMSC-CM, hucMSC-dp-Ex, or hucMSC-Ex for 24 h. Viability of the cells in hucMSC-Ex was higher than those for the control and hucMSC-dp-Ex groups. **c**, **d** HucMSC-Ex promoted HaCaT proliferation in a dose- and time-dependent manner. **e** HucMSC-CM and hucMSC-Ex restored normal HaCaT cell morphology after H_2_O_2_ induction whereas the other treatments could not. **f**, **g** Flow cytometry showed that hucMSC-Ex reduced the fluorescence intensity of HaCaT cells induced with H_2_O_2_. **h**, **i** Flow cytometry showed that hucMSC-Ex inhibited apoptosis of HaCaT cells induced with H_2_O_2_. Compared to control: ^*^P < 0.05 and ^**^P < 0.01; compared to H_2_O_2_:^#^*P* < 0.05 and ^##^*P* < 0.01. Data are representative of three independent experiments (means ± SD)

Paracrine factors play important roles in stem cell-based regenerative medicine. To explore the mechanism by which paracrine factors promote cutaneous wound healing, we exposed HaCaT cells to H_2_O_2_ then cultured them in hucMSCs-CM. The CCK8 assay showed that the viability of the HaCaT cells cultured in hucMSCs-CM was significantly higher than that of the control group (1.48 ± 0.04, 1.20 ± 0.05, and 1.12 ± 0.02 relative to the control group at 24 h, 48 h, and 72 h, respectively) (Fig. 5a). We derived exosomes from hucMSCs by ultracentrifugation and compared their effects on HaCaT cell proliferation with those of the hucMSCs-CM, hucMSCs-dp-Ex, and DMEM/F12 control groups. The hucMSCs-Ex significantly enhanced HaCaT cell proliferation (control vs. hucMSCs-Ex: 1 vs. 1.69 ± 0.03; *P* < 0.05) as did hucMSCs-CM (control vs. hucMSCs-CM; 1 vs. 1.46 ± 0.07; *P* < 0.05). On the other hand, hucMSCs-dp-Ex exhibited no significant efficacy in enhancing HaCaT cell proliferation (*P* > 0.05) (Fig. 5b). Moreover, the viability of HaCaT cells cultured in hucMSCs-Ex increased with a dosage in the range of 125–1000 ng mL^−l^ (0.89 ± 0.05, 0.99 ± 0.04, 1.15 ± 0.04, and 1.48 ± 0.03 relative to the control group) (Fig. 5c). Over time, however, the efficacy of hucMSCs-Ex at attenuating HaCaT cell death decreased, and there was no significant difference from the control after 48 h (Fig. 5d). Phase-contrast microscopy showed that the cell in the hucMSCs-Ex group is better than H_2_O_2_ group (Fig. 5e). Flow cytometry disclosed that the ROS fluorescence intensity in the hucMSCs-Ex group was significantly lower than that of the H_2_O_2_ group (H_2_O_2_ vs. hucMSCs-Ex 2080.44 ± 68.2 vs. 567.17 ± 54.8; *P* < 0.01). Similar results were obtained for the hucMSCs-CM group (H_2_O_2_ vs. hucMSCs-CM 2080.44 ± 68.2 vs. 886.18 ± 63.98; *P* < 0.01) (Fig. 5f, g). The percentage of apoptotic cells for Annexin V-positive cells (AV^+^/PI^-^), in the hucMSCs-Ex group was significantly lower than that of the H_2_O_2_ group (H_2_O_2_ vs. hucMSCs-Ex 21.23 ± 2.84 vs. 4.53 ± 1.07; *P* < 0.01). The percentage of apoptotic cells for Annexin V-and PI positive cell (AV^+^ and PI^+^), in the hucMSCs-Ex group significantly reduced (hucMSCs-Ex vs. H_2_O_2_ 8.67 ± 2.85 vs. 29.75 ± 2.8; *P* < 0.05). This finding was comparable to that determined for the hucMSCs-CM group (AV^+^/PI^-^ cells: H_2_O_2_ vs. hucMSCs-CM 21.23 ± 2.84 vs. 7.25 ± 2.07; *P* < 0.01; AV^+^ and PI^+^cells: H_2_O_2_ vs. hucMSCs-CM 29.75± 2.8 vs. 10.01 ± 2.07; *P* < 0.01 (Fig. 5h, i). Nevertheless, neither the ROS fluorescence intensity nor the proportion of apoptotic cells significantly differed between the H_2_O_2_ group and the hucMSCs-dp-Ex group (*P* > 0.05).

### HucMSC-Ex promoted HaCaT cell migration

**Fig. 6:**
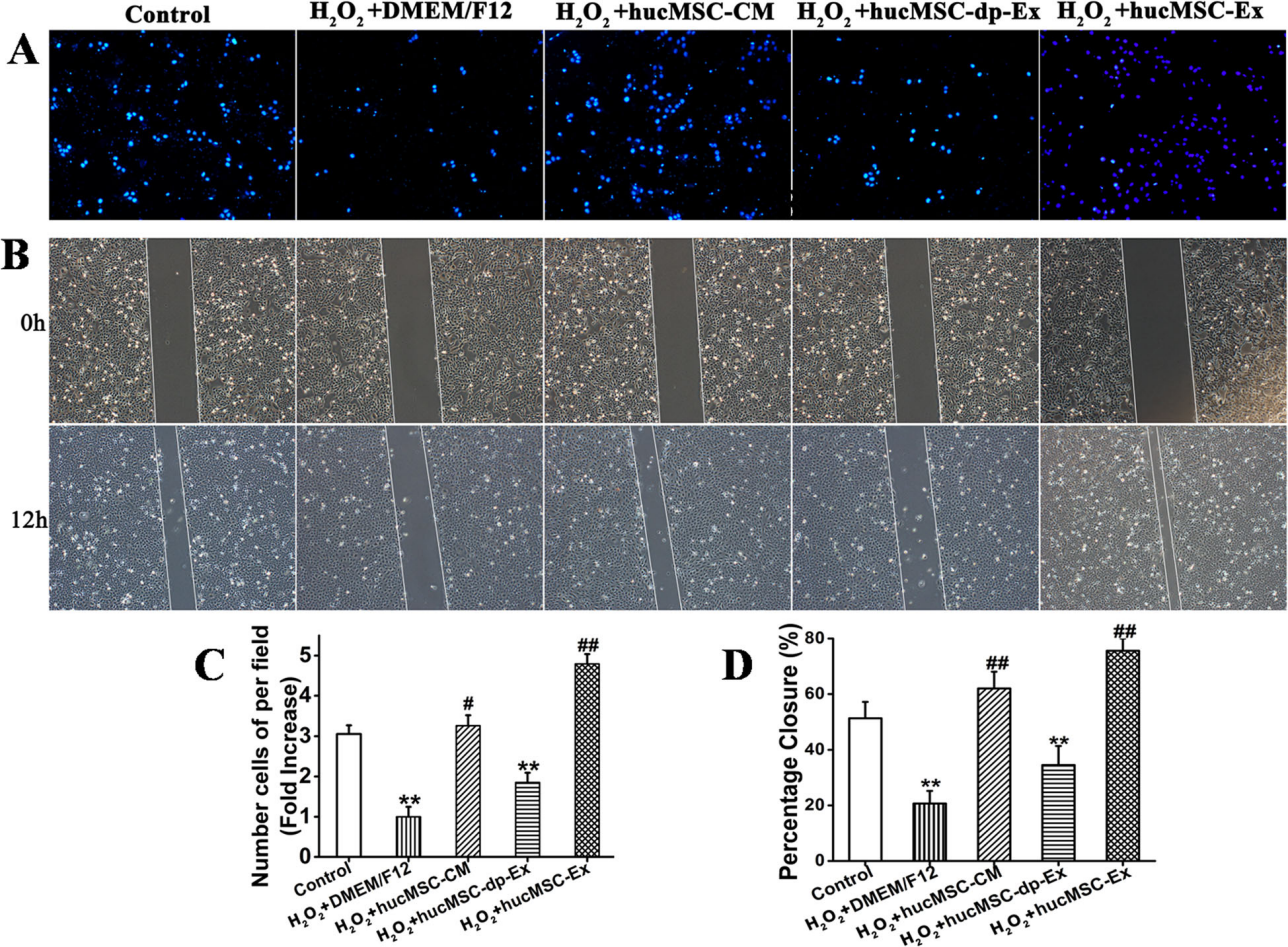
HucMSC-Ex promoted HaCaT cell migration of after H_2_O_2_ induction. HaCaT cells were treated with H_2_O_2_ then cultured in DMEM/F12 (H_2_O_2_ group), hucMSC-CM, hucMSC-dp-Ex, or hucMSC-Ex for 24 h. Control group: normal HaCaT cells. **a**, **c** Transwell cell migration assay showed that the hucMSC-CM and hucMSC-Ex treatments promoted HaCaT cell migration from the upper to lower chambers (*n* = 3; **P < 0.01). **b**, **d** HaCaT cells were treated with various culture media and subjected to a wound-healing assay for 12 h. Percentage closure of wounded areas was measured. The recovery of wound areas was higher in the hucMSC-Ex group than the PBS group (*n* = 3; **P < 0.01). Scale bar = 200 μm. Compared to control: ^*^P < 0.05 and ^**^P < 0.01; compared to H_2_O_2_:^#^*P* < 0.05 and ^##^*P* < 0.01. Data are representative of three independent experiments (means ± SD)

Keratinocyte migration is a critical step in cutaneous wound healing. To explore the effects of hucMSC-Ex on cellular migration, HaCaT cells were pretreated with H_2_O_2_ then incubated with hucMSC-CM, hucMSC-dp-Ex, or hucMSC-Ex for 24 h. The Transwell assay revealed that incubation with hucMSC-Ex or hucMSC-CM significantly increased the numbers of migrated cells relative to the hucMSC-Ex-dp (3.26 ± 0.26 (hucMSC-CM) vs. 3.05 ± 0.22 (control) (*P* < 0.05); (4.5 ± 0.2 (hucMSC-Ex) vs. 3.05 ± 0.22 (control) (*P* < 0.05); 1.9 ± 0.02 (hucMSC-Ex-dp) vs. 21 ± 5.2 (control) (*P* < 0.05)) (Fig. 6a, c). The cell scratching assay showed that the recovery of wound areas of hucMSC-Ex and hucMSC-CM were larger than that for hucMSC-Ex-dp group (62 ± 6 (hucMSC-CM) vs. 21 ± 5 (control) (*P* < 0.05); 76 ± 4 (hucMSC-Ex) vs. 21 ± 5 (control) (*P* < 0.05); 35 ± 6 (hucMSC-Ex-dp) vs. 21 ± 5.2 (control) (*P* < 0.05)) (Fig. 6b, d).

### HucMSCs-Ex attenuated H_2_O_2_ induces HaCaT cell death through suppression of AIF nuclear translocation and PARP-1 activation

**Fig. 7:**
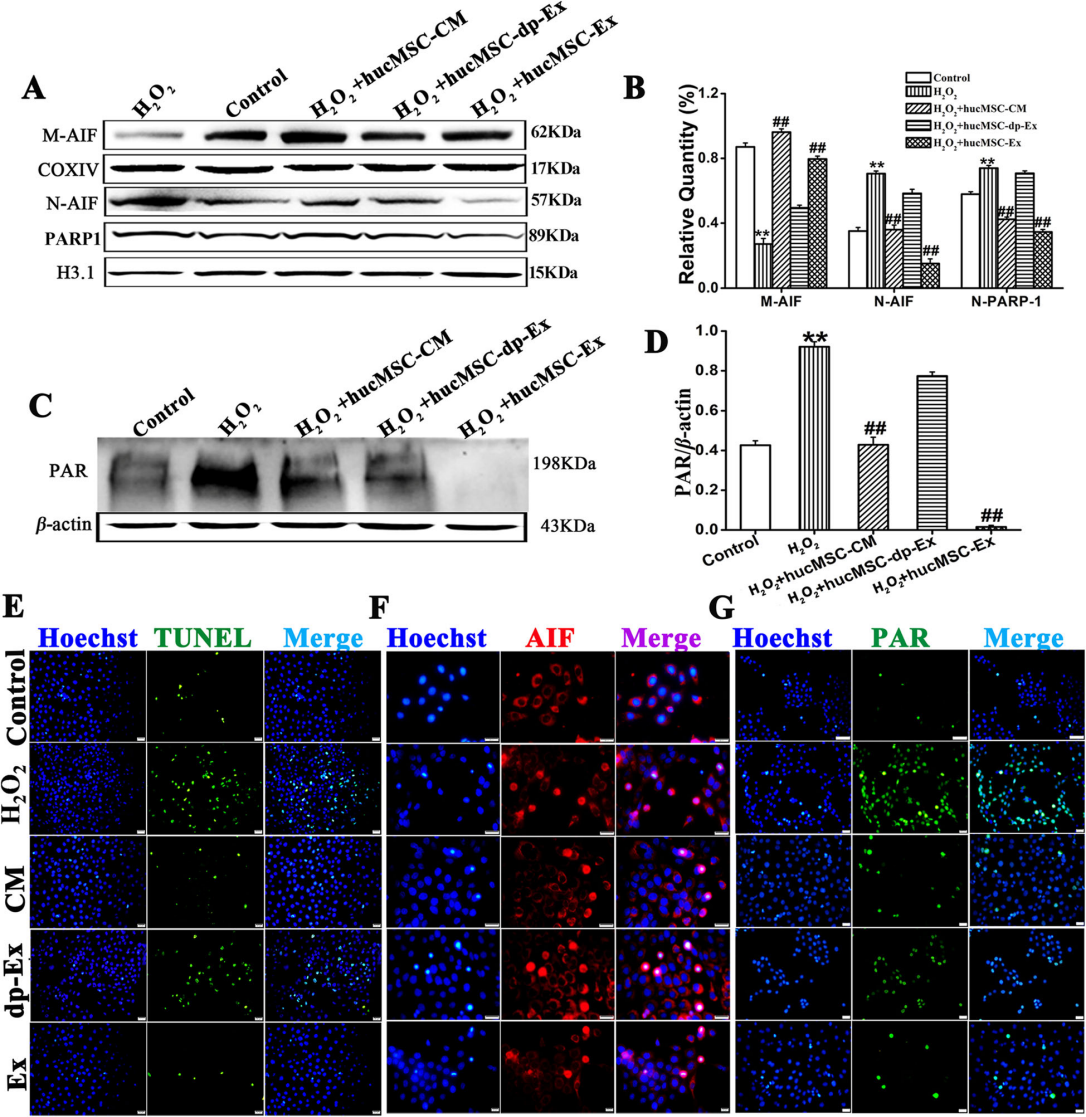
HucMSC-Ex mediates PARP-1 and AIF signaling to inhibit H_2_O_2_-induced cell death. **a** Western blot of nuclear AIF and PARP-1 and mitochondrial AIF expression in HaCaT cells treated with hucMSC-CM, hucMSC-dp-Ex, or hucMSC-Ex for 24 h after H_2_O_2_ induction. **b** Mitochondrial AIF in the hucMSC-dp-Ex-treated HaCaT cells decreased while that for the hucMSC-ex-treated HaCaT cells increased (*n* = 3; **P < 0.01). Nuclear AIF and PARP in the hucMSC-dp-Ex-treated HaCaT cells were higher than those in the hucMSC-ex-treated HaCaT cells (*n* = 3; **P < 0.01). PARP activation in response to the addition of various culture media to the HaCaT cells as detected by western blot using anti-PAR. **c**, **d** PAR in hucMSC-Ex-treated HaCaT cells decreased compared to that for the other treatments (*n* = 3; ***P < 0.001). **e–g** Representative immunofluorescence images and (**h**) quantitative analyses of TUNEL (green), AIF (red), and PAR (green) expressed in HaCaT cells after the addition of various culture media. Blue indicates the nucleus and pink indicates AIF translocation into the nucleus. Scale bar = 200 μm. Compared to control: ^*^P < 0.05 and ^**^P < 0.01; compared to H_2_O_2_:^#^*P* < 0.05 and ^##^*P* < 0.01. Data are representative of three independent experiments (means ± SD)

As our above data showed that H_2_O_2_ induced HaCaT cell death was mediated by AIF nuclear translocation and PARP activation signals. We would like to know if hucMSC-Ex attenuated H_2_O_2_ induce HaCaT cell death was through suppression of AIF nuclear translocation and PARP-1 activation or not. Western blot assay showed compared with the control group, H_2_O_2_ insult significantly reduced mitochondrial AIF expression (*P* < 0.01) but increased nuclear AIF and PARP/1 expression (*P* < 0.01). Compared with H_2_O_2_ group, hucMSC-Ex or hucMSCs-CM significantly reduced nuclear AIF and PARP/1 expression (*P* < 0.01). However, similar to H_2_O_2_ group, hucMSCs-dp-CM did not significantly change mitochondrial AIF expression and nuclear AIF and PARP/1 expression (Fig. 7a, b). In addition, H_2_O_2_ insult increased cytosol PAR accumulation and pretreatment with hucMSC-Ex, hucMSCs-CM significantly reduced PAR accumulation in the cytosol in HaCaT cells (*P* < 0.01) (Fig. 7c, d).

Tunnel staining showed after treatment with H_2_O_2_, the percentage of tunnel positive cells abruptly increased, significantly higher than the control group (H_2_O_2_ vs control, 25.91 ± 1.21 vs 5.61 ± 0.18, *P* < 0.01). However, pretreatment of HaCaT cells with hucMSC-Ex or hucMSCs-CM significantly reduced H_2_O_2_ induced HaCaT cell death (H_2_O_2_ vs hucMSC-Ex, 25.91 ± 1.21 vs 4.88 ± 0.32, *P* < 0.01; H_2_O_2_ vs hucMSCs-CM, 25.91 ± 1.21 vs 6.89 ± 0.27, *P* < 0.01), in contrast to hucMSCs-dp-Ex in that hucMSCs-dp-Ex did not show any effects in reducing H_2_O_2_ induced HaCaT cell death (H_2_O_2_ vs hucMSCs-dp-Ex, 25.91 ± 1.21 vs 20.98 ± 0.42, *P* > 0.05). Immune fluorescence staining showed that after treatment with H_2_O_2_, the percentage of nuclear AIF-positive cells abruptly increased, significantly higher than the control group (H_2_O_2_ vs control, 43.59 ± 0.68 vs5.96 ± 0.57), with remarkable PAR accumulation in the nucleus. However pretreatment with hucMSC-Ex or hucMSCs-CM, the number of nuclear AIF-positive cells abruptly decreased, being significantly lower than the H_2_O_2_ group (H_2_O_2_ vs hucMSC-Ex,43.59 ± 0.68 vs 15.23 ± 0.42, *P* < 0.01. H_2_O_2_ vs hucMSCs-CM, 43.59 ± 0.68 vs 18.62 ± 0.41, *P* < 0.01) and with less PAR accumulation in the nucleus. In agreement with tunnel staining, hucMSCs-dp-Ex did not show any effects in reducing the percentage of nuclear AIF-positive cells (H_2_O_2_ vs hucMSCs-dp-Ex, 43.59 ± 0.68 vs 41.05 ± 0.46, *P* > 0.05) and nuclear PAR accumulation in H_2_O_2_-treated HaCaT cells (Fig. 7e–h).

### HucMSC-Ex dramatically enhanced cutaneous wound healing, skin barrier formation, and angiogenesis, and reduced scar formation

**Fig. 8:**
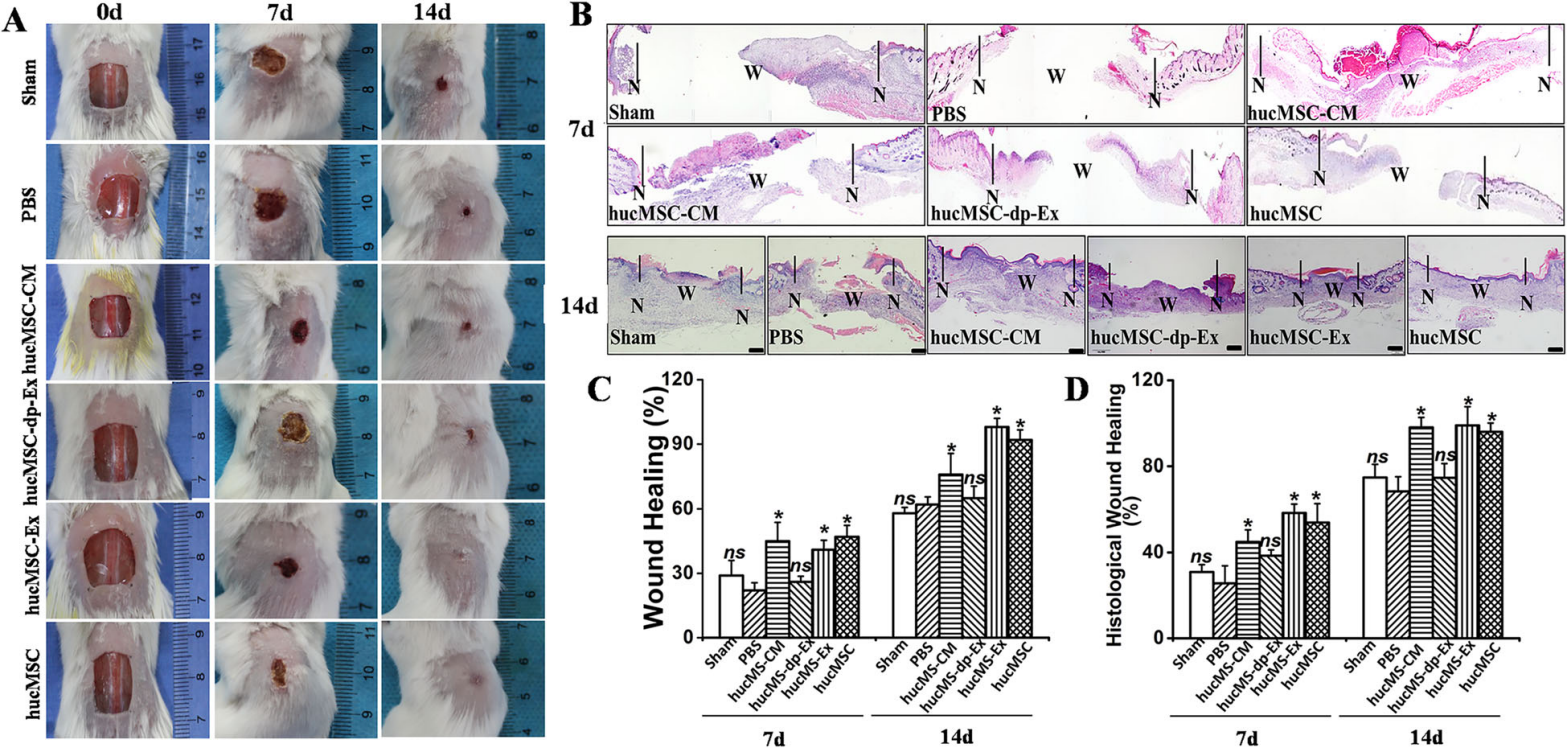
Efficacy of hWJ-MSC in mouse skin wound closure. **a** Gross view of mouse excisional wound splinting model after transplantations of control vehicle medium (sham), PBS, hucMSC-CM, hucMSC-dp-Ex, hucMSC-Ex, or hucMSC at day 0, days 7, and day 14. **b** H&E staining of wound sections treated with PBS, hucMSC-CM, hucMSC-dp-Ex, hucMSC-Ex, and hucMSC at days 7 and 14 after the operation. **N**, normal region; **W**, wound region. Scale bar = 200 μm. **c** Wound measurement in each mouse group; *n* = 6 per group. *P < 0.05 for treatment with hucMSC-CM, hucMSC-Ex, and hucMSC vs. PBS; *P > 0.05 for sham and hucMSC-dp-Ex vs. PBS at days 7 and 14. **d** Proportion of histological wound healing; *n* = 6 per group. *P < 0.05 for treatment with hucMSC-CM, hucMSC-Ex, and hucMSC vs. PBS; *P > 0.05 for sham and hucMSC-dp-Ex vs. PBS at days 7 and 14

To investigate and compare the efficacies of hucMSC-Ex, hucMSC, hucMSCs-CM, and hucMSCs-dp-CM at enhancing skin wound healing, they were separately injected into excised full-thickness mouse skin. Gross inspection showed that hucMSC-CM, hucMSC-Ex and hucMSC injection significantly increased wound closure at day 7 (45.23 ± 4.8% (hucMSCs-CM) vs. 22.35 ± 3.6% (PBS); *P* < 0.01; 41.12 ± 5.2% (hucMSC-Ex) vs. 22.35 ± 3.6% (PBS); *P* < 0.01; 47.23 ± 4.9% (hucMSC) vs. 22.35 ± 3.6% (PBS), *P* < 0.01; 26.31 ± 2.3% (hucMSCs-dp-Ex) vs. 22.35 ± 3.6% (PBS), *P* > 0.01. At day 14: 76.23 ± 8.1% (hucMSCs-CM) vs. 62.41 ± 2.6% (PBS), *P* < 0.05; 98.41 ± 1.9% (hucMSC-Ex) vs. 62.41 ± 2.6% (PBS); *P* < 0.01; 92.23 ± 2.6% (hucMSC) vs. 62.41 ± 2.6% (PBS); *P* < 0.01; 68.42 ± 3.5% (hucMSCs-dp-Ex) vs. 62.41 ± 2.6% (PBS); *P* > 0.01 (Fig. 8a, c).

H&E staining indicated that hucMSC-Ex and hucMSC injection significantly enhanced re-epithelialization in the excisional skin wound at day 7 compared to the PBS group. In contrast, the hucMSC-Ex-dp group had no significant effect (69.1 ± 4.3% (hucMSC-Ex) vs. 32.63 ± 3.5% (PBS); 57.91 ± 5.2% (hucMSC) vs. 32.63 ± 3.5% (PBS); *P* < 0.05; 39.3 ± 2.6% (hucMSC-Ex-dp) vs. 32.63 ± 3.5% (PBS); *P* > 0.05. At day 14: 94 ± 4.2% (hucMSC-Ex) vs. 75.3 ± 3.5% (PBS); 87.91 ± 5.2% (hucMSC) vs. 75.3 ± 3.5% (PBS); *P* < 0.05; 79.63 ± 2.6% (hucMSC-Ex-dp) vs. 75.3 ± 3.5% (PBS); *P* > 0.05 (Fig. 8b, d).

**Fig. 9:**
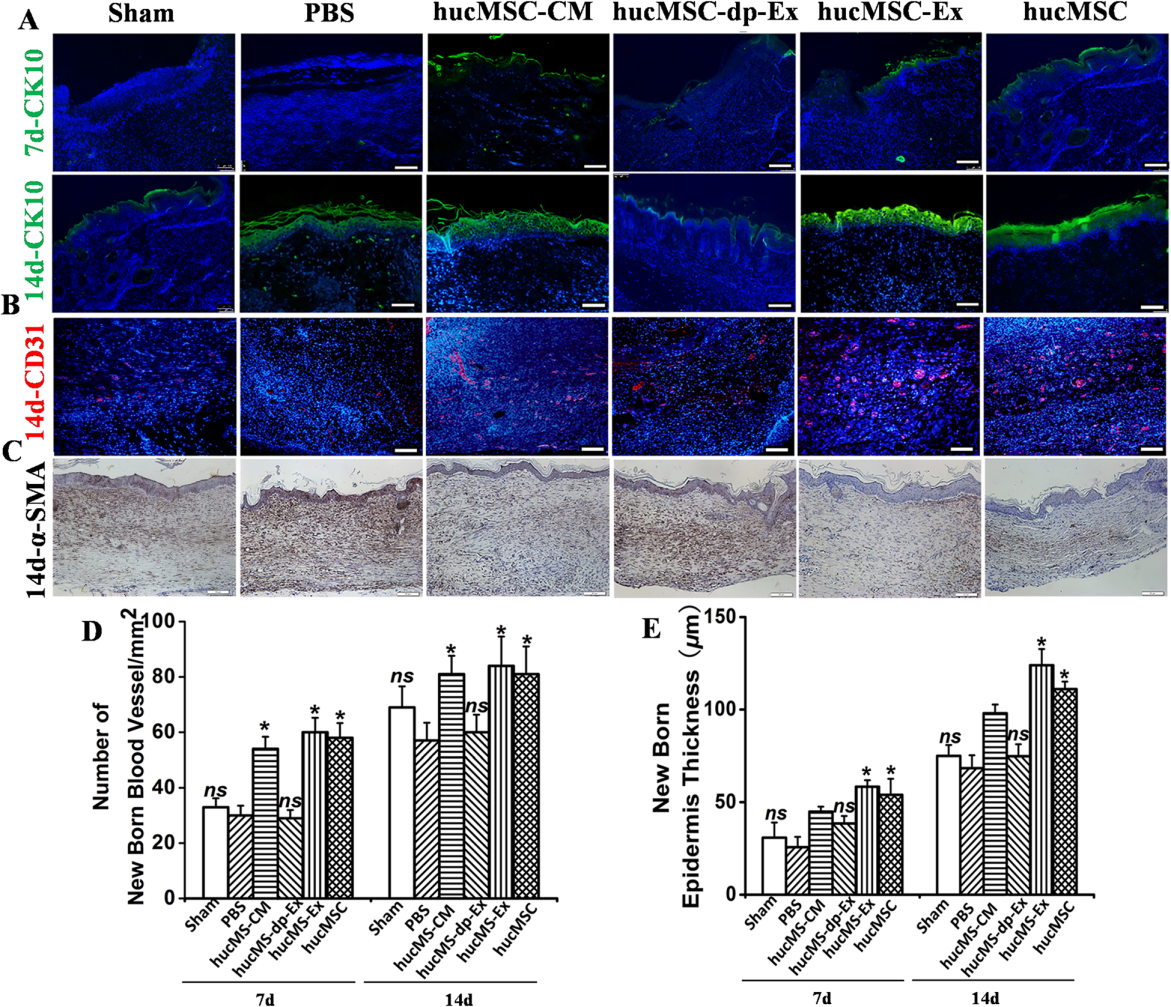
HucMSC-Ex suppresses scar formation and enhances angiogenesis in a full-thickness skin wound model. **a** Wound histology after immune fluorescence staining. Tissue sections obtained from the wound area at days 7 and 14 after various injections were stained with antibodies against cytokeratin 10 (green). Scale bar = 200 μm. **b** Tissue sections obtained from the wound area at day 14 after various injections were stained with antibodies against CD31 (red). Scale bar = 200 μm. **c** α-SMA staining of wound sections treated with hucMSC-CM, hucMSC-dp-Ex, hucMSC-Ex, or hucMSC at day 14. Scale bar = 200 μm. **d** Quantitative analysis of the number of blood vessels in **b**. *n* = 3 per group. *P < 0.05 compared with the PBS group. **e** Quantitative analysis of the thickness of the new epidermis in **a**. *n* = 3 per group. *P < 0.05 compared with the PBS group

Immunohistological staining showed that hucMSC-Ex and hucMSC injection significantly upregulated CK10, CD31 and downregulated α-SMA 14d post-skin wounding (Fig. 9a–e). The optical density for α-SMA in the skin wound tissue of the hucMSC-Ex-dp group was significantly higher than those for the hucMSC and hucMSC-Ex groups. Thus, hucMSC-Ex enhanced cutaneous wound healing, re-epithelization, and angiogenesis and reduced scar formation.

## Discussion

In this study, we demonstrated that subcutaneous hucMSC-Ex injection promoted rapid wound closure, re-epithelialization, and new blood vessel formation, and reduced scar formation. Thus, it significantly enhanced skin wound healing.

To explore the mechanism by which hucMSC-Ex stimulated the healing of skin wounds, a skin cell injury model was created by exposing HaCaT cells to H_2_O_2_. Peroxide induced HaCaT cell death through mitochondrially mediated caspase-dependent- and caspase-independent apoptosis signaling pathways. Previous reports indicated that the caspase-independent apoptosis pathway is called PARP-1-dependent cell death or *parthanatos* [27–29]. It is biochemically distinct from classically defined cell death pathways and comprises rapid PARP-1 activation, early PAR accumulation, mitochondrial depolarization, early nuclear AIF translocation, loss of cellular NAD and ATP, and late caspase activation [30, 31]. H_2_O_2_-induced HaCaT released ROS which injures DNA and activates PARP-1 [32, 33]. Our results showed that H_2_O_2_ induced AIF nuclear translocation, PARP-1 hyperactivation, and PAR accumulation. H_2_O_2_ decreases mitochondrial membrane permeability, releases cyt C, activates caspase-3, and stimulates cell apoptosis. When the broad-spectrum caspase inhibitor Z-VAD.fmk and the PARP-1 inhibitor DPQ were added to the HaCaT cells, the apoptosis rate in the DPQ group was significantly lower than that of the Z-VAD.fmk group. Moreover, whereas Z-VAD.fmk could not inhibit AIF translation to the nuclei, DPQ was able to do so. Caspase activation is a hallmark of apoptotic cell death but does not appear to play any role in parthanatos as broad-spectrum caspase inhibitors could not protect the cells [34, 35]. Thus, H_2_O_2_-induced HaCaT apoptosis might be mediated via the PARP-1 cell death pathway or parthanatos.

Over the years, MSC-based therapies [36] have been investigated as a means of augmenting the structure and function of damaged or diseased tissues via direct cell replacement. When MSCs are labeled and delivered in vivo, they migrate to tissue injury sites such as brain lesions and cardiac infarcts [37, 38]. However, relatively few MSCs are actually engrafted at these damage sites and most of them are cleared [39]. Paracrine factors rather than transdifferentiation is now considered the predominant mechanism by which MSCs affect tissue repair. Experiments with MSC-conditioned media demonstrated the importance of the paracrine mechanism of hucMSC in the promotion of wound healing [40, 41]. Our previous study demonstrated that hucMSCs enhance mouse skin wound healing [24].

Exosomes are membrane-bound vesicles produced by MSCs. In cell culture, they are released into the ambient medium. They may be critical messengers for cell-to-cell communication [42]. Studies have shown that stem cell-derived exosomes activate resting stem cells in the wound microenvironment by regulating inflammatory and immune responses [43]. In this manner, they proliferate and differentiate into tissue-specific cells, repair damaged tissue cells, promote angiogenesis, improve the local wound microenvironment, and repair and reconstruct tissues. Our results showed that hucMSC-Ex inhibited H_2_O_2_-induced cell death in a time- and dose-dependent manner. Other studies reported that hucMSC-Ex inhibited H_2_O_2_-induced cell death by suppressing AIF translation to the nuclei and subsequent PARP-1 hyperactivation [44]. Thus, hucMSC-Ex may mediate a caspase-independent pathway via AIF and PARP-1 to inhibit repair cells such as fibroblasts and keratinocytes in deep apoptosis. In this way, it accelerates cutaneous wound repair and regeneration. We conducted animal tests to determine whether hucMSC-Ex promotes skin wound healing and confirmed by gross inspection and histological observations that it significantly repaired excisional full-thickness defects. The wound healing rate for the hucMSC-Ex group was significantly higher than those for the hucMSC-dp-Ex and PBS groups. Histologically, hucMSC-Ex enhanced skin re-epithelialization. Immunohistological staining was used to elucidate the mechanism by which hucMSC-Ex promotes cutaneous wound healing. CK14, CD31, and α-SMA staining disclosed that hucMSC-Ex significantly enhanced skin barrier formation and angiogenesis while dramatically reducing scar formation. CK14 is expressed in the normal epidermis [45]; therefore, its upregulation in the wound after hucMSC-Ex exposure means that the treatment improved the integrity of the newly formed epidermis. Vascular endothelial cells express CD31 [46]; thus, we used it to evaluate the number of newly formed blood vessels in the re-epithelialized tissue following hucMSC-Ex treatment. The α-SMA is highly expressed in scar tissue [47, 48]; for this reason, we applied it to confirm its expression in healing skin. Certain reports demonstrated that MSC-Ex may promote skin wound healing by promoting miR-21-3p-mediated angiogenesis and fibroblast function. Other studies proved that exosomes derived from MSCs enhance skin wound healing by inducing NrF2 [49], MALAT1 [50], or Wnt4 [51] protein factors that mediate cell proliferation, angiogenesis, and migration. Here, however, we showed that hucMSC-Ex inhibits skin cell apoptosis via the PARP-1 pathway and participates in tissue repair and reconstruction.

There were several limitations to the present study. First, it was not established whether it is more advantageous to heal skin wounds by using large volumes of simultaneously injected exosomes or small volumes of exosomes administered over a long period of time. Second, the changes in AIF or PARP-1 expression in wound tissues after hucMSC-Ex transplantation and the mechanism by which AIF/PARP-1 regulates target gene expression were not evaluated in the present study. Third, it was not determined which exosome components regulate the caspase-independent apoptosis pathway in wound healing stimulation. We went further and established the exosomal mechanism of action by tracking exosomal interaction with cells in the real time. We then delved deeper and identified the abundant proteins within the exosomes cargo using a proteomics approach and to look for the key protein for wound healing.

In summary, our research indicated that hucMSC-Ex effectively enhances cutaneous wound healing in mice possibly by HaCaT cell activation in the skin. The hucMSC-Ex promoted HaCaT cell proliferation and migration and inhibited H_2_O_2_-induced HaCaT cell death in vitro. In hucMSC-Ex-dependent regulation of HaCaT cell function, the caspase-independent PARP-1 and AIF apoptosis pathway play vital roles as hucMSC-Ex markedly reduces AIF translocation and PARP-1 hyperactivation. Our findings suggest that hucMSC-Ex is a new therapeutic tool for tissue wound healing by inhibiting deep apoptosis of repair cells. Next, maybe we will choose the big animals, such as pig, to experiment that whether the hucMSCs-Ex can also enhance diabetic skin wound healing. After elucidating the mechanism of hucMSCs-Ex for tissue repair, it may contribute to the treatment of clinical patient-related diseases in the future.

## Conclusion

The hucMSc-Ex effectively enhances cutaneous wound healing possibly by activating HaCaT cells in the skin. HucMSC-Ex promotes HaCaT cell proliferation and migration, and inhibits H_2_O_2_-induced HaCaT cell apoptosis in vitro via a caspase-independent pathway in which PARP-1 activation signals AIF release from the mitochondria to the nuclei.

## Data Availability

All data generated or analyzed during this study are included in this published article.

## Abbreviations

AIF: Apoptosis-inducing factor
BSA: Bovine serum albumin
cyt C: Cytochrome C
DMEM: Dulbecco’s modified Eagle’s medium
DMSO: Dimethylsulfoxide
FBS: Fetal bovine serum
GAPDH: Glyceraldehyde-3-phosphate dehydrogenase
H&E: Hematoxylin and eosin
H_2_O_2_: Hydrogen peroxide
HaCaT: Immortalized human keratinocytes
HRP: Horseradish peroxidase
hucMSC: Human umbilical cord mesenchymal stem cells
hucMSC-CM: HucMSC-derived conditioned medium
hucMSC-dp-Ex: Exosomes-deprived hucMSCs-conditioned media
hucMSC-Ex: HucMSCs-derived exosomes
hucMSCs: Human umbilical cord mesenchymal stem cells
MSC: Mesenchymal stem cells
PAR: Poly (ADP-ribose)
PARP-1: Poly (ADP-ribose) polymerase-1
PMSF: Phenylmethylsulfonyl fluoride
